# African Swine Fever in Uganda: Qualitative Evaluation of Three Surveillance Methods with Implications for Other Resource-Poor Settings

**DOI:** 10.3389/fvets.2015.00051

**Published:** 2015-10-28

**Authors:** Erika Chenais, Susanna Sternberg-Lewerin, Sofia Boqvist, Ulf Emanuelson, Tonny Aliro, Emma Tejler, Giampaolo Cocca, Charles Masembe, Karl Ståhl

**Affiliations:** ^1^National Veterinary Institute, Uppsala, Sweden; ^2^Swedish University of Agricultural Sciences, Uppsala, Sweden; ^3^Gulu District Local Government, Gulu, Uganda; ^4^Torslanda Djurklinik, Torslanda, Sweden; ^5^Makerere University, Kampala, Uganda

**Keywords:** participatory epidemiology, smartphone, outbreak investigation, infectious animal diseases, low-income countries, disease detection

## Abstract

Animal diseases impact negatively on households and on national economies. In low-income countries, this pertains especially to socio-economic effects on household level. To control animal diseases and mitigate their impact, it is necessary to understand the epidemiology of the disease in its local context. Such understanding, gained through disease surveillance, is often lacking in resource-poor settings. Alternative surveillance methods have been developed to overcome some of the hurdles obstructing surveillance. The objective of this study was to evaluate and qualitatively compare three methods for surveillance of acute infectious diseases using African swine fever in northern Uganda as an example. Report-driven outbreak investigations, participatory rural appraisals (PRAs), and a household survey using a smartphone application were evaluated. All three methods had good disease-detecting capacity, and each of them detected many more outbreaks compared to those reported to the World Organization for Animal Health during the same time period. Apparent mortality rates were similar for the three methods although highest for the report-driven outbreak investigations, followed by the PRAs, and then the household survey. The three methods have different characteristics and the method of choice will depend on the surveillance objective. The optimal situation might be achieved by a combination of the methods: outbreak detection via smartphone-based real-time surveillance, outbreak investigation for collection of biological samples, and a PRA for a better understanding of the epidemiology of the specific outbreak. All three methods require initial investments and continuous efforts. The sustainability of the surveillance system should, therefore, be carefully evaluated before making such investments.

## Introduction

Animal diseases have negative impacts on markets, trade, economy, and public health at farm and national levels (1, 2). In resource-poor settings, these impacts are especially severe as the animals have multiple roles in the economy, and individual households, as well as the national income, are highly dependent on their livestock (3, 4). Therefore, disease control is important for mitigation of these negative consequences. According to the Food and Agriculture Organization of the United Nations and its “Progressive Control Pathway for Foot and Mouth Disease Control” (5), the first step in achieving control is to understand the disease epidemiology within its local context. This is a valid strategy for most animal diseases, but in low-income countries, this basic understanding is often missing (6). Such epidemiological understanding is normally gained through some form of disease surveillance system (7, 8).

Resource allocation for the entire chain of surveillance is costly. For active surveillance, this chain includes cost-effective planning, sample selection, sampling, transportation, laboratory analysis of biological samples, and epidemiological analysis of the results. Although the activities related to active sampling are not applicable for passive surveillance, planning and analysis of results are still important and must be taken into account, as well as any reactive actions following the results. Paradoxically, the need for surveillance is often greatest where it is most difficult to achieve, i.e., in resource-poor settings (9, 10). In low-income countries, current surveillance systems for endemic diseases are often inefficient and dysfunctional (11–13). Factors contributing to these failures are deteriorating administrative services, continuous budget reductions, and lack of veterinary personnel (6). Lack of infrastructure as well as population and animal registers make surveillance difficult. Furthermore, the surveillance systems in these settings are often not designed with the objective to meet the needs of the common smallholder farmer, but rather to meet trade requirements for other circumstances and accessible by only a minority of commercial farmers (14).

Alternative methods have been proposed for overcoming the challenges of disease surveillance in resource-poor settings. Some of these methods specifically aim to collect surveillance data from populations that are normally not reached by traditional surveillance, such as official reporting via veterinarians. Participatory disease surveillance, stemming from the development of rapid rural appraisals in the 1970s (15–20) comprises a set of methods that have been used for various situations, including emerging diseases (21, 22), endemic diseases (23, 24), and the eradication of rinderpest (25, 26). Lately smartphone-based applications have been developed for different surveillance purposes and stakeholder categories (27–29). Other systems have used regular mobile phone calls (30) or text messages (31, 32). These alternatives to traditional surveillance have been described for a specific context, disease, or issue. Some studies have compared alternative methods to the traditional surveillance (33), but the different alternatives have seldom been evaluated. Despite numerous alternative surveillance methods being piloted, few studies have compared the alternative methods in the same area for the same disease to evaluate their relative performances.

In Uganda, 24.5% of the population lives below the national poverty line, and poverty is more prevalent in the rural areas (34). The country has the largest pig population in east Africa and the most rapidly growing pig population in sub-Saharan Africa (35). Most pigs are kept in smallholder family farms in the rural areas (36, 37). African swine fever (ASF) is one of the biggest hurdles for the development of the pig sector (38, 39). The disease is endemic in the domestic pig population, and there are numerous studies and media reports of outbreaks (37, 40–43). ASF is a notifiable disease in Uganda, meaning that veterinarians are obliged to report outbreaks to the central veterinary authority. Official reports confirm that outbreaks occur almost yearly, but in the last available report from the World Organization for Animal Health (OIE) the disease is marked as “present,” but without any quantitative data (44). The true scale of the endemicity is, thus, unknown.

Passive surveillance is generally considered as the most appropriate form of surveillance for acute infectious diseases with high mortality, such as ASF. However, especially in low-income countries, the disease-detection capacity of passive surveillance systems is hampered by distrust of governmental authorities, low disease awareness, lack of financial compensation, and stigmatization of affected farmers. Thus, there is a need for alternative surveillance methods (7). To control diseases, such as ASF in low-income countries, surveillance alternatives must be assessed and compared. Cost-effectiveness, feasibility, and the capacity to detect the disease are factors to be evaluated. However, these comparisons are not straightforward as the results from different methods reflect many different aspects of the diseases under consideration.

The objective of this study was to evaluate and qualitatively compare alternatives to traditional surveillance for acute infectious diseases in resource-poor settings with particular focus on disease-detection capacity. The study used the case of ASF in northern Uganda as an example and included report-driven outbreak investigations, participatory rural appraisals (PRAs), and a household survey using a smartphone application.

## Materials and Methods

This evaluation compares three different studies carried out in and around the Gulu district in northern Uganda between September 2010 and January 2014. The three studies were consecutively applied in the study area and had different designs and original purposes, each including a different surveillance method. Some villages and/or households were sampled by more than one of the three methods. Outbreaks of ASF were described at the household level (report-driven outbreak investigation and household survey) and the group level (PRA) and were defined differently according to the respective study design.

### Study Area and Study Population

The Gulu district covers 3,449 km^2^ and is divided into 2 counties, 12 subcounties, 70 parishes, and 294 villages (45). The Gulu municipality is divided into five divisions, each holding four parishes. The village is the smallest administrative unit. No formal household or animal registry exists. A human population census was conducted in 2014 and the equivalent for domestic animals in 2008. According to these, the district has 443,733 inhabitants in 87,786 households, including 6,200 pig-keeping households with in total 26,570 pigs (46, 47). The pig production is characterized by a low-input, free-range husbandry system (48). Pig farming is promoted by the government and non-governmental organizations. The prospective study population included all pig-keeping households in the Gulu district. This district was severely affected by an armed conflict between 1986 and 2006 (49, 50), but it is now slowly recovering from the political, social, economic, and military unrest.

### Report-Driven Outbreak Investigations

Between September 2010 and November 2011, reports of ASF outbreaks in and around the Gulu district were investigated by the district veterinary officer (DVO) in Gulu, as previously described (51).

#### Selection of Participants

All reports of disease in pigs characterized by fever and mortality that reached the DVO in Gulu during the study period were included. The affected households were subsequently visited by the DVO.

#### Data Collection

Blood and serum samples from the *vena jugularis externa* were collected from at least one clinically diseased pig where possible; otherwise, the samples were taken from apparently healthy pigs in those households that still had any surviving pigs at the time of visit. Samples were stored overnight in a fridge at the district veterinary office in Gulu before transport to the laboratory. During transport, samples were kept cool with ice in a cooler bag. On arrival to the laboratory serum samples were centrifuged and sera separated, and serum and blood samples stored separately at −20°C until analysis.

After laboratory analysis of the samples, all villages with households with positive results were re-visited by the DVO. During this visit, key informants provided information pertaining to all households that had been affected by the recent ASF outbreak in each village. All these affected households were included in the subsequent part of the study that comprised collection of additional data by household interviews using semi-structured questionnaires. The questionnaires were delivered by the DVO in the local language (Luo) with notes taken in English on a paper copy of the questionnaire. The data collected included household location (GPS coordinates), starting month of the confirmed outbreak, number of pigs that had died or survived the outbreak, and number of pigs at the time of the visit. Questionnaire data were entered into Microsoft Excel^®^ spreadsheets (Microsoft, Redmond, WA, USA) as soon as possible after each visit.

#### Laboratory Investigations

All laboratory analyses were done at the Molecular Biology Laboratory at Makerere University, Institute of Environment and Natural Resources, in Kampala. Outbreaks were laboratory confirmed by detection of ASFV nucleic acids using a commercially available real-time PCR (Tetracore Inc., Rockville, MD, USA) in accordance with manufacturer’s instructions (52).

#### Outbreak Definition

An outbreak of ASF was defined as a household in a village from which ASF had been laboratory confirmed, with the individual, affected households identified by a key informant.

### Participatory Rural Appraisals

Participatory rural appraisals were conducted between September and October 2013, as previously described (53). This complete PRA protocol included questions related to the participants’ knowledge, attitudes, and practices concerning ASF, but our study only included questions regarding ASF outbreaks.

#### Selection of Participants

Selection of participants was based on purposive sampling strategies (16, 19) and included participants from all 43 villages included in the report-driven outbreak investigation and 10 additional villages. All participants were individually invited by the DVO in Gulu via key informants. Present, or historical, pig keeping was an inclusion criteria. Selection of participants from the 43 villages that were included in the report-driven outbreak investigation was not limited to members of households that had participated in that study.

#### Data Collection

Our PRA protocol was adopted from one developed by the International Livestock Research Institute (ILRI) Smallholder Pig Production Value Chain Development (SPVCD) project in Uganda (54). The PRAs were conducted in the local language (Luo) by one facilitator and one note-taker, both of whom were native Luo-speakers and proficient in English. The facilitators and note-takers, who had been trained in participatory methods, research ethics and the protocol prior to the implementation of the PRAs, exchanged roles between the PRAs. Outbreaks were listed and questions relating to disease characteristics (pigs that died, were sick but recovered, or that never were sick) were answered by proportional piling using beans as markers (17, 19, 55). One hundred beans were used and the size of the piles (equal to number of beans) could thus be converted to a percentage for each answer. For all questions in the PRAs, the participants were asked to consider ASF outbreaks during the last two years. Questions regarding outbreaks were asked after the name for the disease in local language and the clinical signs had been established. Information was triangulated within each PRA by cross-checking answers from several questions and, in addition, through key-informant interviews performed at the same time as the PRAs. All data were entered into Microsoft Excel spreadsheets (Microsoft, Redmond, WA, USA) by the first author as soon as possible after each PRA.

#### Outbreak Definition

An outbreak of ASF was defined at the village level, as stated by the PRA participants.

### Household Survey

A survey of pig-keeping households was undertaken in the study area between December 2013 and January 2014. The survey was delivered by 41 community knowledge workers (CKWs). The CKWs were peer-selected local residents affiliated with the Uganda branch of the Grameen Foundation (http://www.grameenfoundation.org), equipped with, and trained in the use of, mobile phones for delivering surveys and extension services. All participating CKWs took part in a refresher training, including technical aspects of the phone, interview techniques, interview and research ethics, as well as the specific questions in this survey before the implementation. The survey consisted of two parts: the first comprises questions related to the respondents’ pig keeping and the second part comprises a poverty index developed by the Grameen Foundation (56). Only the first part of the survey was relevant to, and included, in this study. The full questionnaire is in Supplementary Material.

#### Selection of Participants

Each CKW was assigned one parish, in most cases, the home parish where they also performed their regular extension services. Parishes not covered by any CKWs were excluded from the study after having controlled that all 12 subcounties were covered. The CKW system was not in place in the Gulu municipality, but five parishes from two divisions in the municipality were still included in the study to have a broader representation of households. These divisions were covered by CKWs from other parts of the Gulu district. The urban parishes were chosen by convenience selection. All villages in these parishes were surveyed. The survey included 16 out of the 43 villages that were included in the report-driven outbreak investigations and 22 out of the 53 villages included in the PRAs. In each village, the selection of households was done by convenience selection of the first pig-keeping household, followed by a snowball sample selection technique (the interviewed household indicated the closest neighboring household keeping pigs) for the subsequent households, i.e., not strictly random (57–59). Households were thus not identified or selected based on participation in the other parts of the study. To focus the sampling on the population of interest, “pig keeping” was set as an absolute inclusion criterion. In each village, all pig-keeping households were included in the study, if <20 in total. If the village had more than 20 pig-keeping households, the snowball selection process ended with the 20th household. The respondent in each household was an adult household member that was at home and available at the time of visit, and who had sufficient knowledge of the family’s pig keeping to adequately answer the questions.

#### Data Collection

The surveys were administered through a smartphone-based application. The surveys were displayed on the phones in English and the questions were asked in the local language (Luo). Answers were continuously registered on the phones during the interview. Immediately after each interview, questionnaire data were transferred to a cloud-based server of the mobile network or, if no mobile network was available, saved on the mobile phones and transferred automatically as soon as the mobile network was reached. On the server, data were stored in csv format. The database was accessed using the survey design platform created by the Grameen Foundation. Data were transferred from Grameen Foundation to the first author in Microsoft Excel spreadsheets (Microsoft, Redmond, WA, USA).

#### Outbreak Definition

An outbreak of ASF was defined as high mortality among pigs at the household level as reported by the respondent.

### Data Analysis and Editing

Data manipulations and analysis, including descriptive statistics, were performed in R (60). Medians, 10th and 90th percentiles were calculated.

To assess the level of misclassification in the data from the household survey, especially considering the vague outbreak definition for this method, a spatial cluster analysis was performed. The hypothesis was that if the described mortalities would be caused by ASF, outbreaks would cluster, and conversely, if outbreaks would not cluster the described outbreaks would not be due to an infectious disease, including ASF, i.e., false positives. In brief, a retrospective, purely spatial, Bernoulli model scan statistic (PSBM) was applied (61). PSBM compares observed and expected numbers of cases in circular areas with different radii. For each space window, a likelihood ratio is calculated to identify to what extent the number of cases inside the area is higher than expected. Monte Carlo permutation (*n* = 999) was used to test for the significance level of spatial clusters. PSBM was run in the open source software SatScan™ (62). Cases were households with outbreaks, according to the definition above, and controls were households without such outbreaks.

Several categories of respondents were excluded from the analysis of results from the household survey. First, respondents who had experienced an outbreak, but who stated that none of their pigs had died or been sick (63 respondents) and, second, respondents who did not have any pigs at the start of a described outbreak, but who anyway mentioned pigs that died or fell sick (4 respondents) were excluded from the disease characteristics analysis. Third, for the visualization of outbreaks per month and year in Figure 2, respondents that had failed to mention the month and year of the outbreak, or that had stated a date earlier than the requested two last years (124 additional respondents) were also excluded, and finally, respondents without correct recordings of GPS coordinates (137 additional respondents) were excluded from the spatial cluster analysis.

From the PRAs outbreaks with dates stated earlier than the requested two last years were similarly excluded from the visualization of outbreaks per month and year in Figure 2 (12 outbreaks).

## Results

### Report-Driven Outbreak Investigations

**Figure 1:**
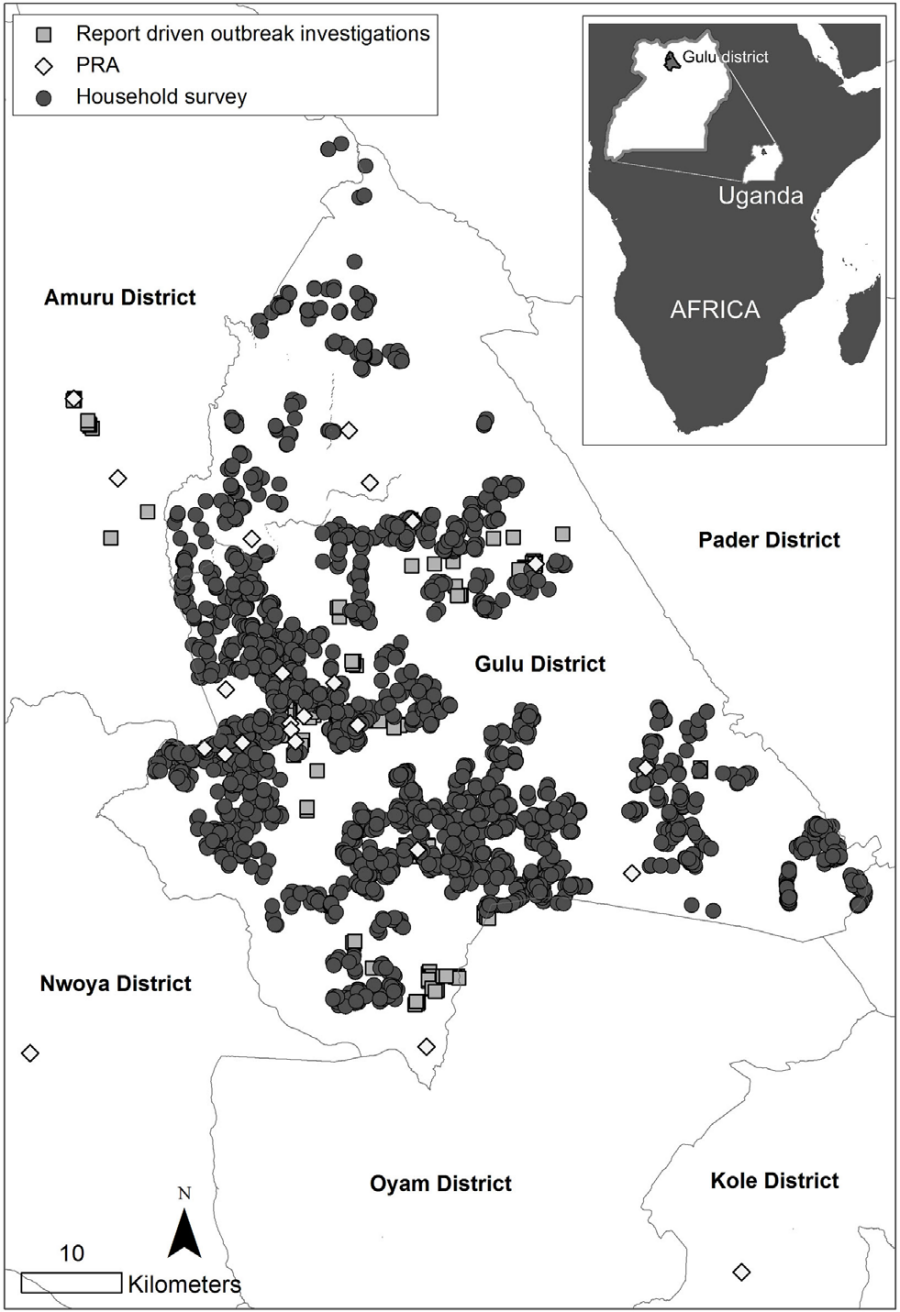
**Geographical distribution of households and participatory rural appraisal series (PRA) in three studies, including three different surveillance methods for African swine fever in smallholder pig production, conducted in northern Uganda 2010–2014**.

**Figure 2:**
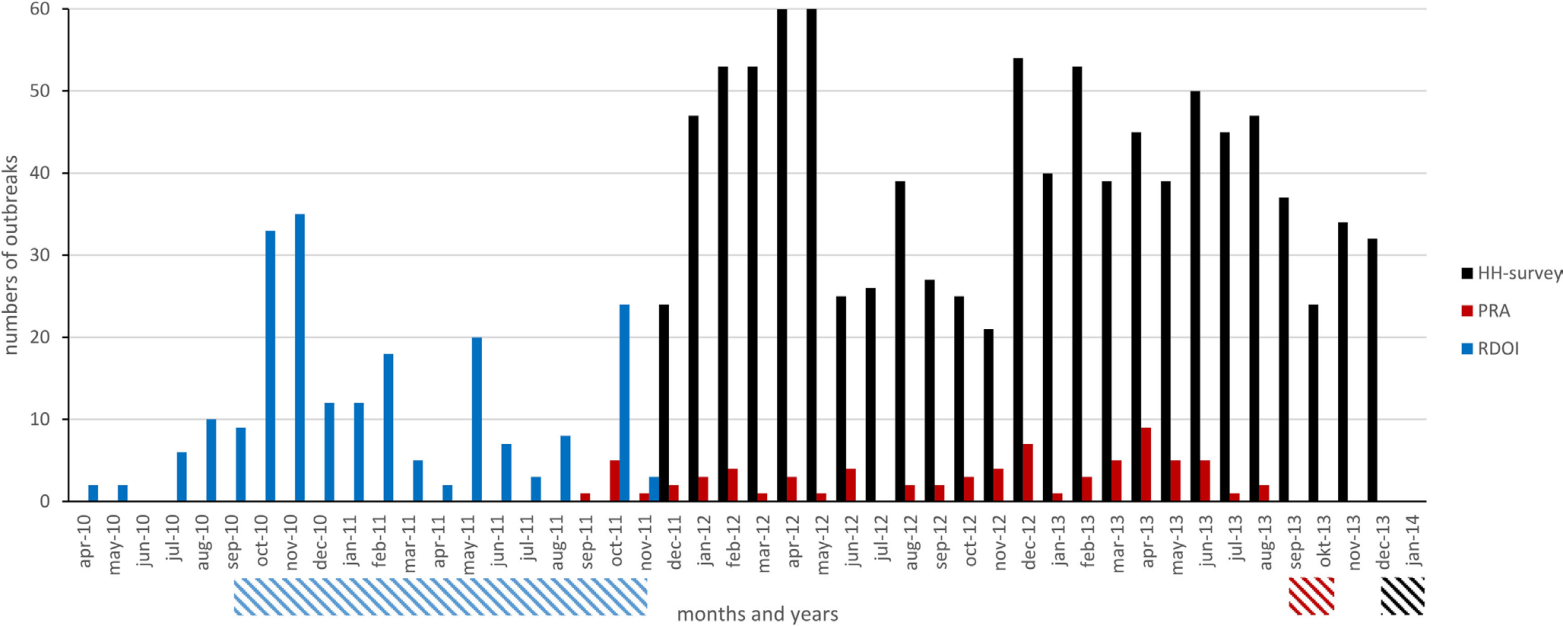
**Number of outbreaks per month (vertical, colored, bars) and time periods for the field work (dashed, horizontal bars) in three studies including three different surveillance methods for African swine fever in smallholder pig production, conducted in northern Uganda 2010–2014**. HH, household; PRA, participatory rural appraisal; RDOI, report-driven outbreak investigations.

Outbreaks were primarily reported from 119 households in 43 villages. All outbreaks were investigated and biological samples taken. ASFV nucleic acids were detected in at least one sample from every investigated village. On the subsequent visit, following the test results, key informants reported a total of 211 affected households from these 43 villages, all of which were interviewed. The geographical distribution of the included households is displayed in Figure 1. In total, 154 (73%) of the respondents were male and 57 (27%) female, see Table 1. During the outbreaks that preceded the interviews, the median proportion of pigs in the included households that had died was 100 (10th percentile 50, 90th percentile 100), see Table 2. All 211 households stated the month and year for the start of the outbreaks. The earliest starting date of an outbreak was April 2010 and the latest November 2011. The number of described outbreaks per month and the time period for the field work is illustrated in Figure 2.

**Table 1:** **Demographic data on participants in three studies including three different surveillance methods for African swine fever in smallholder pig production, conducted in northern Uganda 2010–2014**.

	Report-driven outbreak investigations	PRA	Household survey
No. of participants^a^			
Total	211 (100)	524	4,000 (100)
Male	154 (73)	^b^	2,518 (63)
Female	57 (27)	^b^	1,482 (37)
No. of villages	43	56	218

*^a^(%)*.

*^b^Data not available*.

*PRA, participatory rural appraisals*.

**Table 2:** **Disease estimates and identified outbreaks in three studies including three different surveillance methods for African swine fever in smallholder pig production, conducted in northern Uganda 2010–2014**.

	Report-driven outbreak investigations	PRA	Household survey
No. of outbreaks	211^a^	94^b^	1,225^c^
Affected farmers (%)^d^			
Affected	N/A	79 (46, 97)	N/A
Not affected	N/A	23 (3, 56)	N/A
Household yearly incidence (%)	N/A	N/A	15
Village yearly incidence (%)^e^	8.8	N/A	31
Affected pigs (%)^d^			
Died	100 (50, 100)	80 (50, 96)	67 (20, 100)
Survived	0 (0, 50)	N/A	N/A
Sick but recovered	N/A	0 (0, 9)	0 (0, 50)
Healthy	N/A	16 (3, 46)	0 (0, 50)

*^a^From 43 villages. ASF outbreak defined as a household in a village from which ASF had been laboratory confirmed, with the affected households identified by a key informant*.

*^b^From 44 PRAs including 56 villages. ASF outbreaks defined at the village level, as stated by the PRA participants*.

*^c^Out of 4,000 households interviewed. ASF outbreak defined as high mortality among pigs at the household level as reported by the respondent*.

*^d^Median (10th, 90th percentiles)*.

*^e^Yearly village incidence calculated based on outbreaks starting during a period of 20 months and Gulu district having 294 villages*.

*PRA, participatory rural appraisals*.

### Participatory Rural Appraisals

A total of 524 participants, representing 56 different villages, were included in 44 PRAs. See Figure 1 for the geographical distribution of the PRAs, and Table 1 for the demographic composition of the participants. A total of 94 outbreaks were described in the PRAs, see Table 2. Out of these outbreaks, 74 had occurred in the two years preceding the PRAs. Based on all the 94 described outbreaks, the median proportion of farmers affected by each outbreak was 79% (10th percentile 46, 90th percentile 97), see Table 2. The median proportion of pigs that died during these outbreaks was 80% (10th percentile 50, 90th percentile 96) and very few of the pigs that fell ill survived, see Table 2. The number of described outbreaks per month from September 2011 to September 2013, and the time period for the field work is illustrated in Figure 2.

### Household Survey

**Figure 3:**
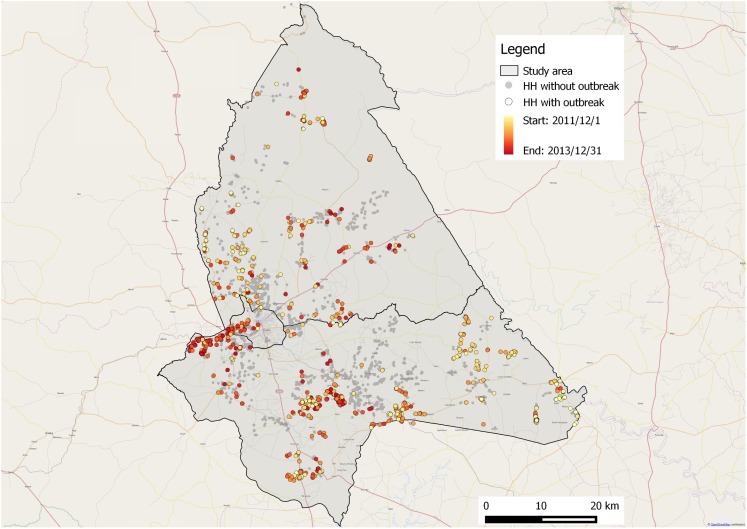
**Spatial distribution of households with and without described outbreaks from a household survey on African swine fever in smallholder pig production, conducted in northern Uganda in December 2013 to January 2014**. HH, household.

In total, 4,000 households were included in the survey, see Figure 1 for their geographical distribution. In total, 2,518 (63%) of the respondents were male and 1,482 (37%) female, see Table 1. The median number of pigs, including piglets, in the interviewed households at the time of the survey was 2 (10th percentile 1, 90th percentile 7.1). In total, 1,225 households reported outbreaks with pig deaths or sickness, see Table 2. Out of these 1,125 households stated the month and year of the outbreaks and 1,101 of those dates were within the two years preceding the study. For 964 out of the 1,101 households, a correct recording of GPS coordinates was available. The spatial distribution of these 964 households, and of the households without any described outbreaks, with correct recording of GPS coordinates (2,669 households), is illustrated in Figure 3. Out of the 964 households with described outbreaks, 60% was included in one of nine significant spatial clusters (*p* < 0.05).

All the 1,225 described outbreaks were included in the analysis of disease characteristics. On average, 15% of the households and 31% of the villages had an outbreak of ASF each year. The households that had experienced outbreaks had in total 7,452 pigs at the start of the outbreaks; the median number of pigs in the affected households was 5 (10th percentile 2, 90th percentile 8). Out of these, a total of 4,722 pigs died in the outbreaks. The median number of pigs that died in each household during an outbreak was 2 (10th percentile 1, 90th percentile 5). According to these results, the median mortality was 67% (10th percentile 20, 90th percentile 100), see Table 2. In total, 1,285 pigs were reported as sick but recovered during the outbreaks, and the median number of pigs that were sick but recovered in each household during an outbreak was 0 (10th percentile 0, 90th percentile 2). In total, 1,394 pigs were never affected in the outbreaks, and the median number of unaffected pigs in each household during an outbreak was 0 (10th percentile 0, 90th percentile 1).

The number of described outbreaks per month from December 2011 to December 2013, and the time period for the field work is illustrated in Figure 2.

## Discussion

All three investigated methods detected a large number of outbreaks. If considering only the number of outbreaks detected per field-day per person, the household survey was the most efficient. However, if data quality aspects were included, this method was less accurate as several outbreaks were not correctly recorded, and had to be excluded from the analysis.

Mortality estimates by the three methods were rather similar, with the highest for report-driven outbreak investigations, followed by the PRAs and the household survey. In east Africa, ASF is mostly associated with high mortality rates, as seen in this study (63, 64), even if outbreaks with lower mortality rates have been described (65–67). In this regard, it is important to note that in resource-poor settings, such as Uganda, mortality rates are difficult to estimate as famers tend to sell or slaughter sick pigs to avoid losses (37). Even if the median mortality rates for the three methods in our study correspond to what is previously known about ASF in east Africa, they were skewed and the intervals between the 10th and the 90th percentiles were wide. This indicates that the estimates were influenced by outlier responses. All three methods in our study estimated mortality rates based on farmer self-assessed disease reports, with the difference that outbreaks had been laboratory confirmed on village level in the report-driven outbreak investigation and that the interviewer in the report-driven outbreak investigation as well as the facilitators in the PRAs were veterinarians, whereas the interviewers in the household survey were not. It is possible that the higher median mortality with these methods, compared to the household survey, are due to the veterinary presence. The veterinarians could have influenced the results by dismissing, and not recording, some outbreaks as non-ASF, whereas some non-ASF cases probably were recorded as outbreaks in the household survey. This is especially plausible given the vague outbreak definition for that survey. However, the majority of outbreaks from the household survey were distributed in accordance with what could be expected for a highly transmissible disease, such as ASF, with several significant spatial clusters identified. This suggests that most of the described mortalities were actually caused by ASF, and adds further credibility to farmer self-assessed disease reports. Outbreaks that were not included in a cluster could have been mortalities caused by something else than ASF, i.e., false positives, true ASF cases that did not spread as would be expected, or outbreaks that were misclassified during the recording process.

Furthermore, the report-driven outbreak investigations were done in close temporal connection to the laboratory-confirmed outbreaks, minimizing the risk for recall bias. By contrast, the PRAs and the household survey asked respondents to consider all outbreaks two  years back in time. In this regard, it is important to emphasize that for all three methods, the resulting disease estimates are just estimates. The uncertainty of the estimates increases in a setting where official animal registration does not exist, where animal owners might be illiterate, where farm records are generally not kept, and where pigs are not individually marked and often free roaming. For the PRAs, the disease estimates were obtained by proportional piling and group consensus. With this tool, it is not the number of beans, but the proportion between the piles (pigs that died, were sick but recovered or that never were sick during each outbreak on village level) that matters. In a context of many uncertainties, this degree of vagueness may make the final answers more useful than misleadingly over-exact measurements. It was further observed that the proportions were changed during the process of reaching a group consensus. The capacity to capture the collective group knowledge, which is sometimes larger than the sum of the knowledge of all the participating individuals, is one of the advantages of the PRA methodology (68). On the other hand, the dependence on group consensus creates the possibility of bias being introduced by dominating participants.

In our previous study (53), a temporal variation in ASF outbreaks was demonstrated based on proportional piling in seasonal calendars from 49 PRAs. The same variation was not obvious in the number of outbreaks per month generated by the three methods included in this study. Applying the same logic as for the disease measures, it can, however, be argued that it is more suitable to use proportional piling than exact dates of outbreaks retrieved at interviews for analyzing diseases dynamics and temporal variation in these contexts. This is especially true if outbreaks are investigated retrospectively.

Report-driven outbreak investigations require that someone contacts the district veterinary office to initiate an investigation. As this step is not included in either the PRA or the household survey, the sensitivity of disease detection by these latter two methods will be higher. The real-time element of the report-driven outbreak investigations avoids recall bias, which may serve to raise both the sensitivity and the specificity of the disease-detecting capacity of the method. The initial investigations of the report-driven outbreak investigations in this study correspond to the current passive surveillance of ASF, outlined in the Ugandan Animal Disease Act (69). DVOs are, however, hindered in fulfilling their statutory tasks due to factors, such as limited budgets and lack of infrastructure for reporting and for making laboratory referrals (13, 70). As found in our study, and as demonstrated by the large number of investigated outbreaks from Gulu during the project period, providing the district veterinary offices with necessary resources can dramatically increase the number of detected and investigated ASF outbreaks.

During the study period (2010–2014), a total of 12 ASF outbreaks were reported from Uganda to the OIE. The concerned authorities detected and recorded more outbreaks, but only laboratory-confirmed outbreaks were reported and all outbreaks occurring within the same district during the same month were grouped as a single outbreak (N. Nantima, Ministry of Agriculture, Animal Industry and Fisheries, personal communication). Bearing in mind the different definition of outbreaks, the three methods included in our study, during a time period ranging from a couple of weeks to 20 months, each discovered many times more ASF outbreaks (in and around Gulu district only) compared to the OIE-reported outbreaks from the entire country. In addition, out of the 10 OIE-reported outbreaks in 2011, 5 were from the Gulu district and detected within the report-driven outbreak investigations of this study.

Only the outbreaks described in the report-driven outbreak investigations of this study were laboratory confirmed, but as many previous studies have demonstrated, farmers are generally able to identify animal diseases that are of importance to them (21, 26, 71, 72). More specifically, the ability of smallholder farmers in northern Uganda to correctly identify outbreaks of ASF has been established by Chenais et al. (53). All PRAs in that study correctly identified at least three clinical signs of ASF and in addition all participants that had previously suffered ASF outbreaks were confident that if a pig showed the clinical signs they (correctly) described, it would be suffering from ASF. Neither classical swine fever nor porcine reproductive and respiratory syndrome (PRRS) is present in the concerned setting. This leaves ASF as one of the few differential diagnoses for infectious pig diseases with very high mortality, further underlining the credibility of farmer-reported ASF outbreaks in Uganda (43). However, farmer reports of more complex issues, such as differentiating “sick due to ASF but recovered” from “sick from something else but recovered,” will naturally have lower specificity. This could have led to miss-classifications, especially in the household survey. All methods defined “ASF outbreaks,” but in different ways. It is important to establish clear case definitions, in English and in local language, before performing any field work in order to minimize the risk for bias. Research including qualitative methods, such as interviews, in non-English speaking communities can introduce additional language and translation bias (73). In our study, this was attended to by having bi-lingual facilitators and by translating the protocols from English to Luo together with all facilitators during the trainings that preceded the PRA and the household survey.

An important factor when comparing and evaluating methods is the attitude and engagement of the respondents, as this affects the quality of the answers, and thus the results (74). Catley and Mohammed (74) observed that participants in PRAs seem to enjoy scoring exercises, in contrast to the informant intolerance reported in studies using structured face-to-face interview techniques. The PRA participants in our study also seemed to enjoy the participatory tools used. Although all three methods included in this study seemed to perform well in their disease-detection capacity, it is important to appreciate a good relationship with the respondents and to avoid any feelings of data retrieval abuse. Participatory methods tackle these issues by involving the participants and letting them guide the discussions (72). However, by letting the participants to choose the subjects without strictly following a questionnaire, it can be difficult to obtain quantitative results that can be evaluated with standard statistical models. This can be overcome by using standardized tools, such as scoring and ranking (22, 71, 72), as was done in this study. Meanwhile, participatory methods effectively capture epidemiological knowledge and in particular qualitative information on interacting sociological, economical, and ecological factors (19, 68, 72). The flexibility offered by the PRA methodology includes instant triangulation that can serve to avoid miss-classifications and the possibility to probe for answers relating to qualitative aspects and causality (19, 72). Some limitations in the PRA methodology are the biases linked to the group dynamics, time requirement, and the difficulties in covering large geographical areas (72, 75). Problems with coverage and remoteness bias will be present for all surveys performed in remote rural areas of low-income countries, unless somehow specifically addressed (75). Through the use of participatory methods, information can be captured from remote areas or populations that would otherwise be inaccessible (19). The use of a broad network of local residents as facilitators or interviewers, such as the CKWs used in the household survey, will also contribute to reducing the remoteness bias.

The objective of this study was not to compare relative costs, but to evaluate and qualitatively compare the methods based on their capacity to detect ASF outbreaks. Nevertheless, the associated costs and feasibility of implementing any given method within a sustainable system must be considered (76). Paterson et al. (76) discuss how the integration of data collection tools into existing information or reporting systems stimulates sustainability of a chosen method. To achieve a sustainable surveillance system after the conclusion of research or development projects, the local institutions must be able to monitor, maintain, and support the setup (77, 78). Several mobile phone surveillance systems have been shown to be highly sustainable with minimal initial investment. A key part in these success stories is the availability of mobile phones, and the opportunity to create two-way information sharing between the reporting farmer or professional and the information receiver (32, 77). Feedback on diagnostic test results and advice on management of the disease problems can both be achieved via a mobile phone-system, for farmers and professionals at different levels in the systems. Such feedback acts as a strong incentive to report (32, 76, 77, 79). The Grameen Foundation CKW system, already in place in Uganda and used for the household survey in this study, could probably be developed into such a sustainable surveillance system for animal diseases if supported by the national institutions and given some initial investments.

The three different methods evaluated have different characteristics and the method of choice will depend on the objective of the surveillance. If rapid and reliable, qualitative epidemiological data from remote areas are needed, PRA seems to fit the purpose best. If there is a need for biological samples and confirmation of diagnoses, outbreak investigations need to be performed. If quick, but not necessarily exact, disease detection in a large area is needed, household surveys using a mobile phone-based system could be a possibility. The optimal solution might even be a combination of the three methods: outbreaks detected in real-time via a smartphone interactive surveillance system, outbreak investigation teams sent to identified areas to take biological samples (if needed), and PRAs performed to define the epidemiology of each specific outbreak. If compensation-measures are available, PRAs can in addition identify beneficiaries.

It could be argued that the different surveillance methods in our study, performed in different studies, with different study designs, at different times, with partially different study participants and on different levels (household and group) cannot be accurately compared, and that this study disqualifies as an attempt to compare apples and oranges (33). Despite these limitations, the inability of current systems in low-income countries to achieve adequate disease surveillance and control of important animal diseases (11) makes it important to attempt such comparisons. The endemic situation in the study area extenuates some of the limitations in study rigor as outbreaks will certainly occur regularly. That is, even as the true number of outbreaks will have varied during the study period, it can be assumed that (many) outbreaks did occur in the study area during the duration of each individual study. The study design including three individual studies, and consecutively applying three different surveillance methods, thus made sure that several, and different, outbreaks were included. As the true number of outbreaks in the area during the study period is unknown this kind of study cannot compare the sensitivity, specificity or individual disease-detection capacity of the different surveillance methods. However, the evaluation of each method made in this study shows that ASF outbreaks can be efficiently detected using farmer reports and that real-time large scale surveillance can be done using a smartphone interactive surveillance system.

Many alternatives to traditional surveillance are presently being piloted, and these must be evaluated to stimulate progression. Policy makers need sound evaluations to make good decisions on how to reform surveillance with the ultimate goal of mitigating the negative consequences of animal disease. In order to achieve this goal, it is important to make use of existing material, such as the data included in this study, even if imperfect, and maybe even incomplete.

## Conclusion

The three methods demonstrated a disease-detecting capacity above that of official reporting, establishing that ASF outbreaks can be efficiently detected using farmers’ reports. The mortality estimates derived from the three methods corresponded reasonably well with each other, with the report-driven outbreak investigations scoring highest, then the PRAs followed by the household surveys. The three methods have different qualities, and the method of choice will depend on the objective of the surveillance. All three methods require at least some initial investments and could be combined for maximized flexibility and efficiency.

## Ethical Statement

The District Veterinary Office, under the Ministry of Agriculture, Animal Industry and Fisheries (MAAIF) has the official mandate to carry out investigations to animal disease in the country. Such investigations can include various methods of information collection, such as sampling of animals and interviews with animal owners. All handling of animals including sampling was carried out, or overseen, by District Veterinary Office staff in accordance with their national mandate. All interviews of animal owners was carried out, overseen, or approved by, District Veterinary Office staff in accordance with their national mandate. For this reason, no additional ethical clearance was deemed necessary for any project activities. All participants, in all the three methods, were informed that the study was a research-project, about the purpose of the study, the voluntary aspect of participation and how their personal integrity would be protected prior to their participation. Oral informed consent was assured by all participants in the report-driven outbreak investigations and the PRA. For the household survey, consent was included in the first question of the questionnaire.

## Author Contributions

EC designed the study. EC designed the questionnaires for the PRAs and the household survey. EC performed the fieldwork for the PRAs and assisted in the fieldwork for the household survey. EC drafted the manuscript. SS-L, SB, UE, and KS assisted in design of the study, the questionnaires for the PRAs and the household survey, and the draft of the manuscript. KS and CM assisted in designing the questionnaire for the report-driven outbreak investigations and in the field work for that study. TA and ET designed the questionnaire and performed the field work for the report-driven outbreak investigations. KS and CM supervised the laboratory analysis. GC performed the spatial cluster analysis. All authors read and approved the final manuscript.

## Conflict of Interest Statement

The authors declare that the research was conducted in the absence of any commercial or financial relationships that could be construed as a potential conflict of interest.
