# Supplementary material for: African Swine Fever in Uganda: Qualitative Evaluation of Three Surveillance Methods with Implications for Other Resource-Poor Settings
Source: Front Vet Sci. 2015 Oct 28;2:51. doi: 10.3389/fvets.2015.00051 (PMC4673915; doi:10.3389/fvets.2015.00051)
Supplement: Supplementary file 1 [file data_sheet_1.pdf]

NVI Baseline (en)

Variable Name	Question Text	Saved Value
q1	Hello, my name is .... and I am a Community Knowledge Worker (CKW) with Grameen Foundation (GF). GF in partnership with National Veterinary Institute of Sweden (the Department of Disease Control and Epidemiology) is conducting a rapid household census in Gulu districts. The project intends to provide a regional estimate of the share of households currently keeping pigs by herd size and structure that will serve as the basis for later extrapolating study results. All information collected in this study is confidential and there are no known risks associated with participating in this survey. Your participation is voluntary and all personal identifier like your name, contacts address will not appear anywhere in the report. Do you agree to participate in the interview?	1 Yes<br>2 No
q2	Date	User selected date
q3	Respondent's name	User entered text
q4	District	1 Gulu
q5	Sub-county	1 Awach<br>2 Bungatira<br>3 Paicho<br>4 Unyama<br>5 Palaro<br>6 Patiko<br>7 Bobi<br>8 Koro<br>9 Lakwana<br>10 Lalogi<br>11 Odek<br>12 Ongako<br>13 Layibi<br>14 Bardege<br>15 Pece<br>16 Laroo<br>17 Other
q6	If other, please specify	User entered text

q7	Parish	User entered text
q8	Village	User entered text
q9	Respondent's gender	1 Male<br>2 Female
q10	Do you currently have pigs in your Household?	1 Yes<br>2 No
q11	What kind of pigs do you have?	1 Sows<br>2 Boars<br>3 Piglets/Growers
q12	Number of Sows	User entered integer
q13	Number of Boars	User entered integer
q14	Number of Piglets/Growers	User entered integer
q15	Has your household had outbreaks of disease with pigs dying suddenly during the past two years?	1 Yes<br>2 No
q16	Hidden from user	
q17	State Date (month and year)	User entered text
q16	Hidden from user	
q17	State Date (month and year)	User entered text
q18	How many pigs died?	User entered integer
q19	How many pigs were sick but recovered?	User entered integer
q20	How many pigs were never affected?	User entered integer
q21	Has your village had outbreaks of disease with pigs dying suddenly during the past two years?	1 Yes<br>2 No
q22	Hidden from user	
q23	State Date (month and year)	User entered text
q22	Hidden from user	
q23	State Date (month and year)	User entered text
q24	How many members does the household have?	

		1 Six or more<br>2 Four or five<br>3 Three<br>4 Two<br>5 One
q25	Do all children ages 6 to 18 currently attend school (government, private, NGO/religious, or boarding)?	1 Not all attend<br>2 All attend government schools<br>3 No children ages 6 to 18<br>4 All attend, and one or more attend a private, NGO/religious, or boarding school
q26	What is the highest grade that the female head/spouse completed?	1 No female head/spouse<br>2 P.5 or less, or none<br>3 P.6<br>4 P.7 to S.6<br>5 Higher than S.6
q27	What is the major construction material of the roof?	1 Thatch, straw, or other<br>2 Iron sheets, or tiles
q28	What is the major construction material of the external wall?	1 Un-burnt bricks, mud and poles, thatch/straw. timber, stone,

		burnt bricks with mud, other<br>2 Burnt bricks with cement, or cement blocks
q29	What is the main source of lighting in your dwelling?	1 Firewood<br>2 Tadooba, or other<br>3 Paraffin lantern, or electricity (grid, generator, solar)
q30	What is the type of toilet that is mainly used in your household?	1 Bush (none)<br>2 Covered pit latrine (private or shared), VIP latrine (private or shared), uncovered pit latrine, flush toilet (private or shared), or other
q31	Does any member of your household own electronic equipment (e.g., TV, radio, cassette, etc.) at present?	1 Yes<br>2 No
q32	Does every member of the household have at least two sets of clothes?	1 Yes<br>2 No
q33	Does every member of the household have at least one pair of shoes?	1 Yes<br>2 No
q34	Capture GPS Location	User captured location coordinates
